# Quasi-BIC Mode Lasing in a Quadrumer Plasmonic Lattice

**DOI:** 10.1021/acsphotonics.1c01416

**Published:** 2022-01-07

**Authors:** Rebecca Heilmann, Grazia Salerno, Javier Cuerda, Tommi K. Hakala, Päivi Törmä

**Affiliations:** † Department of Applied Physics, Aalto University School of Science, P.O. Box 15 100, Aalto, FI-00 076, Finland; ‡ Institute of Photonics, University of Eastern Finland, FI-80 101 Joensuu, Finland

**Keywords:** plasmonics, nanophotonics, surface plasmon resonance, bound-state-in-continuum, lasing

## Abstract

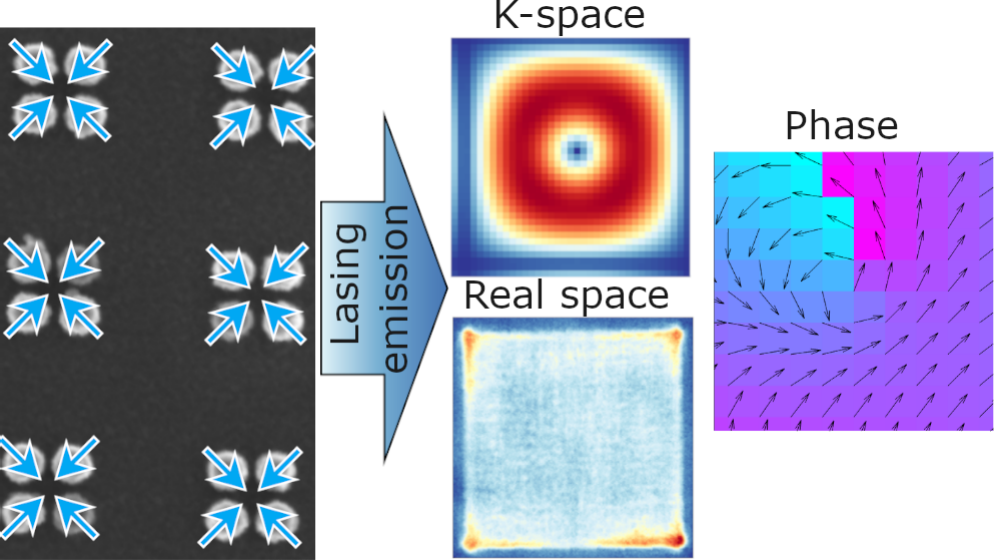

Plasmonic lattices of metal nanoparticles have emerged as an effective platform for strong light–matter coupling, lasing, and Bose–Einstein condensation. However, the full potential of complex unit cell structures has not been exploited. On the other hand, bound states in continuum (BICs) have attracted attention, as they provide topologically protected optical modes with diverging quality factors. Here, we show that quadrumer nanoparticle lattices enable lasing in a quasi-BIC mode with a highly out-of-plane character. By combining theory with polarization-resolved measurements of the emission, we show that the lasing mode has a topological charge. Our analysis reveals that the mode is primarily polarized out-of-plane as a result of the quadrumer structure. The quality factors of the out-of-plane BIC modes of the quadrumer array can be exceedingly high. Our results unveil the power of complex multiparticle unit cells in creating topologically protected high-*Q* modes in periodic nanostructures.

Arrays of metal nanoparticles host dispersive plasmonic–photonic modes called surface lattice resonances (SLRs). The dispersion can be tailored, yielding ultrafast dynamics from the strong near fields of plasmonic nanoparticles. Such plasmonic lattices combined with a gain medium display strong light–matter coupling, photon and polariton lasing, and Bose–Einstein condensation.^1−6^ Thus far, simple unit cells of only one or two particles have been applied, leaving the rich potential of particle complexes^7−10^ untapped. Here we show that a quadrumer nanoparticle array enables lasing in a bound-state-in-continuum (BIC) mode with unique characteristics arising from the multiparticle unit cell.

BICs appear both for classical and quantum waves.^11−14^ They are localized states embedded in the continuum of extended modes and, although resonant, are completely decoupled from them. BICs have diverging quality factors *Q* as light cannot couple out from them; in practice, BICs are observed by coupling them to leakage channels. These features make BICs appealing for applications in lasing, filtering, or sensing.^15−20^ BICs are associated with a quantized topological charge, arising from a nontrivial vorticity in polarization of the far-field radiation;^21,22^ the modes are robust since they can disappear only when annihilated with other BICs of opposite topological charge. BICs appear dark in the outgoing radiation due to the impossibility of defining a propagation wave-vector at the origin of the topological charge.^23,24^ Hence, BICs are an invigorating addition to the field of topological photonics^25^ where unidirectional propagation and topological lasing have already been demonstrated.^26−33^

In this work, we combine a two-dimensional lattice built from a quadrumer structure with a gain medium. Under optical pumping, we experimentally observe the strongest emission at the corners and edges of the sample. By theoretical analysis of the isolated quadrumer, combined with numerical analysis of the electromagnetic fields both in finite and infinite systems, we identify BIC modes in an infinite lattice, and quasi-BIC in a finite one. Comparison of polarization-resolved lasing images to the numerical calculations reveals the lasing occurs in a quasi-BIC mode. The mechanism of coupling the BIC light to far field is in our system provided by the edges of the sample. We observe a nontrivial winding of the polarization field both in the experiments and simulations, indicating a nonzero topological charge. We find that the polarization of the BIC lasing mode is mainly out-of-plane, even when the single nanoparticle is just 50 nm high. In the few previous studies of BICs with out-of-plane polarization,^17,34,35^ the out-of-plane character was already present at the single nanoparticle level. In contrast, our case reveals that the structure of the quadrumers plays a crucial role in determining the polarization pattern of the modes. From infinite lattice simulations, we find the lasing BIC mode to have a *Q*-factor of several thousands, significantly higher than typical SLR modes (*Q* ∼ 100–300,^36,6^ with a record observation of 2340 at telecom frequencies^37^). The lasing emission from the finite-lattice quasi-BIC mode has *Q* ∼ 1400, typical for state-of-the-art plasmonic lasing. Hence, the full potential of the underlying BIC remains to be exploited. We also discover that the momentum dependence of the BIC *Q*-factor dramatically depends on losses.

In summary, our work shows that nanoparticle complexes offer a new promising route for the design of topologically protected high-*Q* modes for lasing and related phenomena. The potential of complex unit cells in the design of quasi-BICs has been studied in the nonlasing regime.^38^ Our work experimentally demonstrates lasing in such a system. Remarkably, we find lasing in a mode whose out-of-plane character stems from the quadrumer structure of the unit cell. Furthermore, our experiments show that the edges of the sample can function as a leakage mechanism of a quasi-BIC. As an interesting outcome from simulations, we found that losses may cause a major qualitative change of the momentum-dependence of the quasi-BIC *Q*-factor.

## Methods

**Figure 1:**
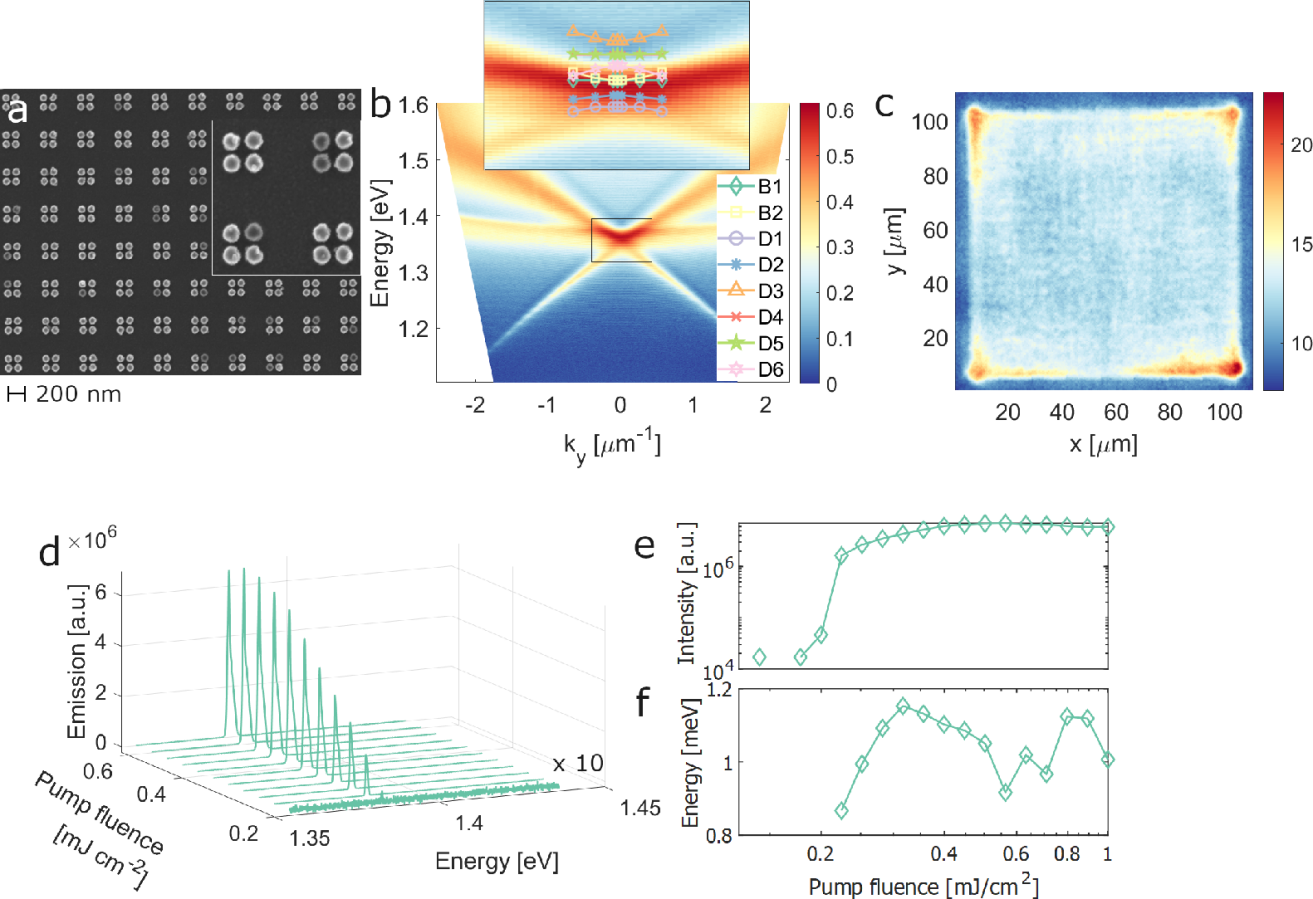
Characterization of the array and lasing experiments. The quadrumer array with a unit cell size of 590 nm and a particle diameter of 100 nm, in dye molecule solution (IR-140, 10 mM), is pumped with 800 nm left circularly polarized (LCP) femtosecond laser pulses. No polarization filter is applied. (a) SEM image of the array; inset shows a magnified view of 2 × 2 unit cells. (b) Dispersion of the bare (no dye molecules) sample given by transmission measurements; inset shows dispersions of the modes calculated by finite-element simulations. (c) Real space image of the array combined with gain (dye) at the lasing regime. (d) Emission spectra and (e) peak intensity as functions of the pump fluence; the lasing mode shown in (c) corresponds to the fluence 0.564 mJ/cm^2^. (f) Full width at half-maximum (fwhm) of the lasing peak as a function of pump fluence. Due to low intensity, we are not able to determine the fwhm below the threshold.

We fabricate arrays of cylindrical gold nanoparticles, organized in a square Bravais lattice with a basis consisting of a quadrumer, that is, four nanoparticles arranged in a square geometry. The unit cell size (period) is *p* = 590 nm, and the distance between the particles in the unit cell is *a* = β*p*/2 nm. In Figure 1a, we show a scanning electron microscope (SEM) image of the lattice with β = 0.5, including the edge termination in the inset. The system supports surface lattice resonances (SLRs), hybrid modes of the metallic surface plasmon resonances and diffracted orders of the lattice.^4,6^ The energy dispersion *E*(*k*_*y*_) of the SLRs is shown in Figure 1b, where *k*_*y*_ is the in-plane momentum in the *y*-direction, and the color scale indicates the intensity of the far-field radiation. The energy of the Γ-point at *k*_*y*_ = 0 is determined by the unit cell size. The inset in Figure 1b shows the dispersion of the modes calculated from finite-element simulations, which are in good agreement with the experimentally observed dispersion. The phenomena we show are not highly sensitive to the choice of structural parameters; their effect is discussed in the Supporting Information.

In the lasing experiments, the arrays are immersed in a fluorescent dye solution (IR-140) and the system is pumped with a left circularly polarized (LCP) 100 fs laser pulse with 800 nm center wavelength (right circular polarization leads to the same results; circular polarization was used to preserve the symmetry of the *x* and *y* directions). The pump mainly couples to the dye molecules and excites them (weak excitation of the broad single particle resonance is also possible). The pump spot covers the entire area of the array, including edges. To characterize the lasing action, we split the emitted light to simultaneously perform angle, energy and position resolved measurements (Supporting Information, Figure S3); the observed real and momentum space images thus correspond to a single lasing mode, as confirmed by a single narrow peak in the energy measurement.

## Results and Discussion

Figure 1c shows the lasing mode, measured as a real space or position-resolved image of the array above the lasing threshold. Interestingly, the emission is stronger on the edges and corners of the array than in the bulk. Figure 1d–f shows the emission spectra, peak intensity, and full width at half-maximum of the lasing peak as a function of pump fluence. From Figure 1d,e, a clear threshold behavior is observed, with an increase in emission of 2 orders of magnitude. The lasing peak appears at *k*_*y*_ = 0 and is located at 1.382 eV and has a line width of 1 meV, yielding a *Q*-factor of 1382.

**Figure 2:**
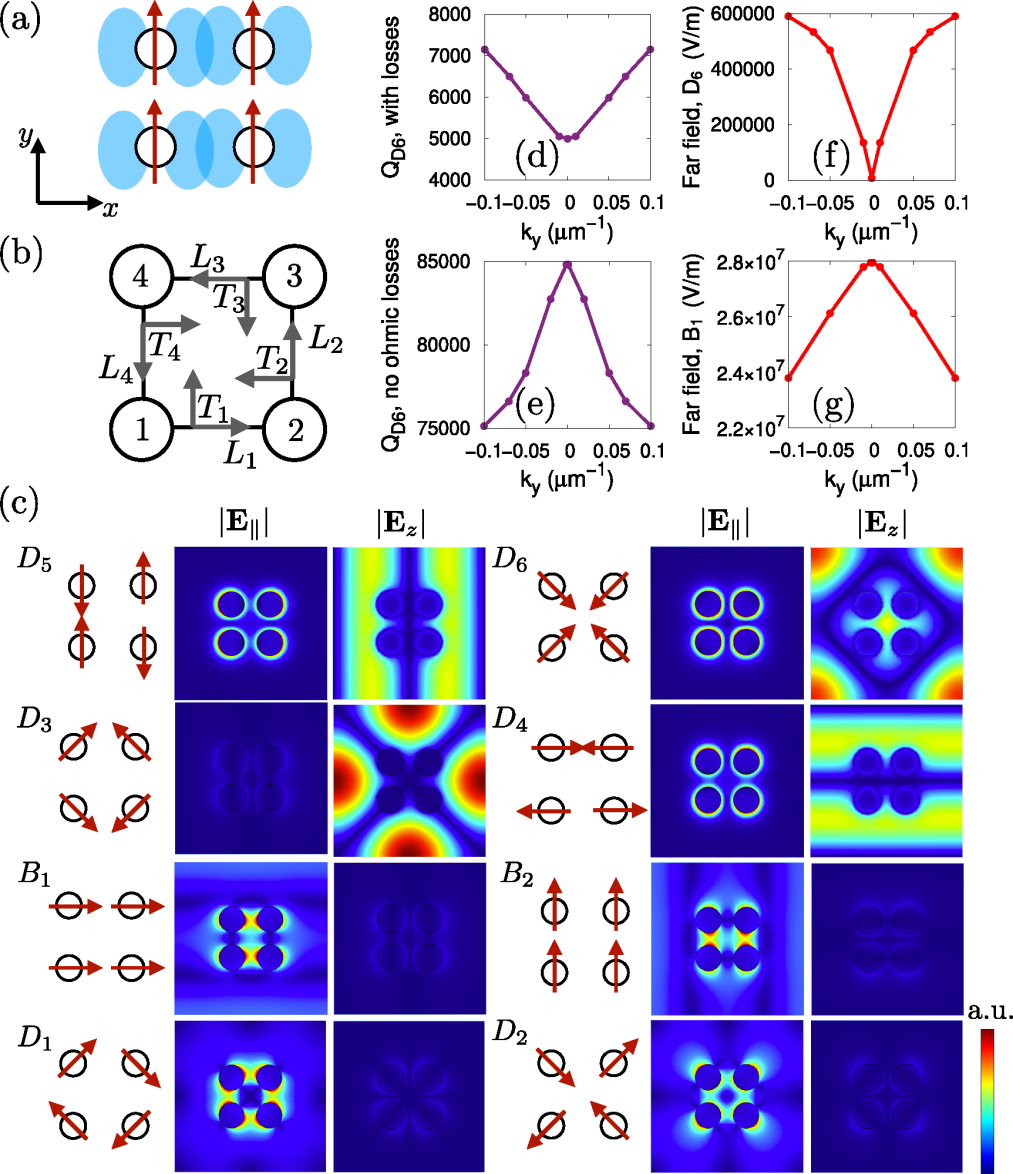
Theoretical analysis of the quadrumer lattice modes. (a) Schematic of the polarization dependence of the electric field coupling in a single quadrumer: for nanoparticles that are vertically polarized, the coupling is stronger in the horizontal direction than in the vertical one. (b) Diagram of the notation used for calculating the normal modes. (c) Normal modes of a single quadrumer, where arrows represent the polarization orientation at each site. We also show the in-plane (|**E**_∥_|) and out-of-plane (|**E**_*z*_|) electric fields of the corresponding modes as calculated from finite-element simulations at the Γ-point (*k*_*x*_ = *k*_*y*_ = 0) on a square lattice of quadrumers. Two bright modes *B*_1,2_ and six dark modes *D*_1,2,3,4,5,6_ are found. The bright modes *B*_1,2_ and two of the dark modes *D*_1,2_ are mainly polarized in the lattice plane, while the remaining dark modes *D*_3,4,5,6_ are primarily polarized out-of-plane, with only a small in-plane field. (d) *Q*-factors of the mode *D*_6_, including a version (e) for which ohmic losses have been removed. (f) Far-field average in-plane amplitude of mode *D*_6_, including losses, and (g) of mode *B*_1_, as obtained from FEM simulations.

We theoretically analyze the modes of the system to identify their polarization pattern and further characterize the lasing action. We consider a simple model of an isolated quadrumer and compute the normal modes of oscillation of the charge distribution, obtaining the dipole moment for each nanoparticle. The coupling in this simple model depends on the polarization orientation with respect to the directions (longitudinal *L*_*i*_ and transverse *T*_*i*_) of the neighboring particles, see Figure 2a,b, and Supporting Information for the details. We find eight modes in total, of which two are bright, labeled *B*_1,2_ and six are dark *D*_1,2,3,4,5,6_, shown in Figure 2c; the arrows represent the dipole moment at each site.

A complementary analysis based on finite-element simulations (FEM) is performed on an infinite two-dimensional lattice of quadrumers, which accounts for the radiative and dispersive nature of the system. At the Γ-point, this calculation yields the same eight modes of the isolated quadrumer model shown in Figure 2c, with a color scale that compares the norm profile of the in-plane () with the out-of-plane (|**E**_*z*_|) components of the various electric fields of the modes. We notice that four modes, namely, *B*_1,2_ and *D*_1,2_, are mainly polarized in-plane with respect to the lattice plane, while the remaining four (*D*_3,4,5,6_) are mostly polarized out-of-plane, with only a small in-plane field component. The structure of these modes is in excellent agreement with those obtained from the isolated quadrumer model.

The FEM analysis also yields the mode dispersion, which is reported in the inset of Figure 1b, for comparison with the experimental measurement. The dispersion of the visible in-plane modes strongly agrees with the experiment, but the out-of-plane dark modes *D*_3,4,5,6_ are not visible. Modes that are polarized in-plane can be dark at some specific in-plane momentum **k** due to symmetry, such as the symmetry-protected BICs, while modes that are completely polarized out-of-plane are dark at every **k**, not just at the high-symmetry points. However, a small nonzero in-plane component makes the coupling to the in-plane polarization possible, albeit too weak to be seen in the experimental dispersion. Nonetheless, a symmetry-protected BIC at Γ point can have either an in-plane or an out-of-plane character.^39,40^

We then calculate the *Q*-factors of the corresponding modes, shown in Figure S1 in the Supporting Information. We find that the *Q*-factors of the in-plane polarized dark modes *D*_1,2_ peak at *k*_*y*_ = 0, compatibly with a symmetry-protected BIC. The out-of-plane polarized modes *D*_4,6_ have very high *Q*-factors at the Γ-point, but the *k*_*y*_-dependence yields a minimum instead of a maximum. We attribute this behavior to the high dissipation losses featured by the metallic nanoparticles, which have a maximal effect at the Γ-point where the SLR modes have a strongly plasmonic character (away from the Γ-point they have a larger photonic component). To show this, we compare the simulation of the mode *D*_6_ in Figure 2d, with its counterpart when the ohmic losses are manually set to zero. The resulting *Q*-factor in Figure 2e features a maximum at *k*_*y*_ = 0, as expected for a BIC. The losses thus drastically change the *Q*-factor dependence on the in-plane momentum. Note, however, that these considerations were based on the *Q*-factor defined as the ratio of stored energy to the power loss, which accounts for both near-field and far-field radiation. Alternatively, the *Q*-factor can be estimated from the radiation emitted by the sample that is detected in the far field. In Figure 2f we show (in the case that includes ohmic losses) that the mode *D*_6_ has a negligible far-field emission at the Γ-point as compared with other *k*_*y*_ values: this behavior is typical for a BIC, as it corresponds to an infinite lifetime at *k*_*y*_ = 0 when defined via the line width of far-field radiation. This is in contrast to the far field emission for the bright mode *B*_1_ shown in Figure 2g, which is maximal at *k*_*y*_ = 0. Analysis of the out-of-plane component of the Poynting vector confirms that the dark modes emit less to the far field than the bright ones (Supporting Information, Figure S6). Mode *D*_6_ thus corresponds to a BIC mode in the lossless, infinite lattice case. It becomes a quasi-BIC mode due to both ohmic losses and finite sample size; the former makes the *Q*-factor finite in the near field but the far field intensity is still zero, while the latter also induces far-field radiation. In total, the system hosts four quasi-BIC, namely, *D*_1,2,3,6_.

**Figure 3:**
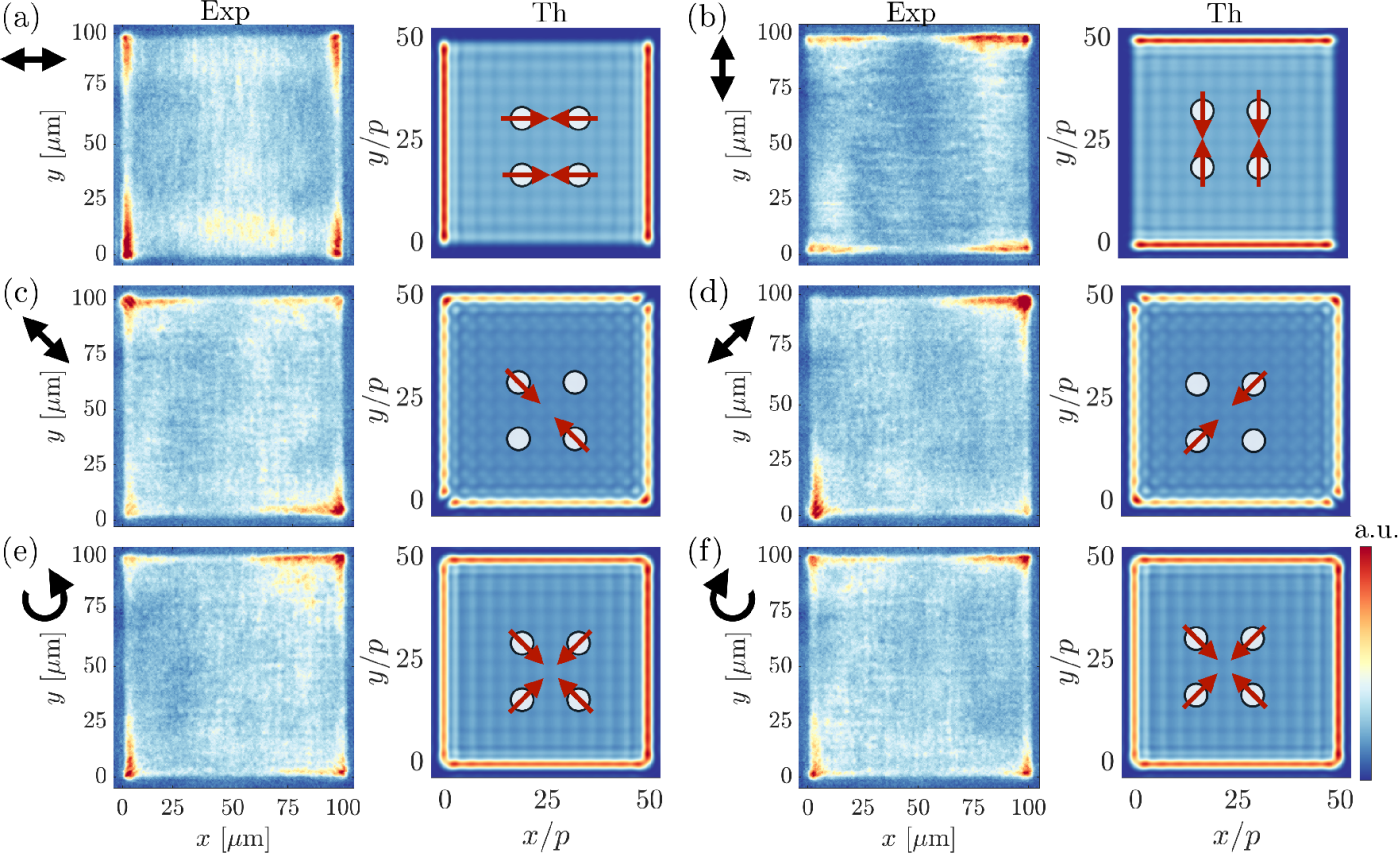
Identification of the lasing mode through polarization measurements. Real-space intensities of the lasing mode with different polarization filters in the detection path. The experimental lasing images are for the case of LCP pumping at a fluence of 0.563 mJ/cm^2^; the theoretical images are based on a numerical calculation of mode *D*_6_ for a finite lattice of 50 × 50 unit cells based on field radiated by in-plane dipoles, including the effect of small losses of about 0.02 in units of the dipole moment. The colors (red high, blue low) represent the simulated intensity of radiation that one would observe in the far field. On top of the simulated 50 × 50 unit-cell field intensity distribution, we depict schematically one (enlarged) unit cell showing the dipole orientations when different polarizers are applied. (a) Horizontal, (b) vertical, (c) antidiagonal, (d) diagonal, (e) right-circular, and (f) left-circular polarization filter.

We now analyze the polarization profile of the lasing mode from real space experiments. The sample is pumped with an LCP pump, and different polarizers are applied in the detection path to filter only a given polarization component of the emission. The pump excites the dye molecules which have a large Stokes shift, that is, the energy difference between emission and absorption is large, and the emission from the molecules couples to the modes present in the nanoparticle array. Since the molecules are in the near-field of the particles, coupling to dark modes is possible. Figure 3 shows the real space intensity images of the array emission above the lasing threshold, filtered by a polarizer oriented as indicated by the black arrows in the figure. We compare these lasing mode images with the theoretical images of mode *D*_6_ in a finite lattice of 50 × 50 unit cells, filtered with the same given polarizer; the inset also shows the corresponding dipole orientation obtained from the isolated quadrumer model. The *E*_*x*_ and *E*_*y*_ field components of the modes are given in Supporting Information, Figure S5. The theoretical images are obtained as the diffraction pattern generated by the vectorial (*x* and *y* polarizations) electric field generated by the in-plane dipole at each nanoparticle, as in ref (41), and include the effect of losses as a small imaginary part, which is 0.02 in units of the dipole moment. These numerical calculations describe the fields that can radiate off from the sample plane to the far field (see Supporting Information).

In all cases of Figure 3, we see that the emission of the lasing mode is stronger at the boundaries of the sample with respect to the bulk. This feature can be theoretically explained as a destructive interference between the electric fields radiated by in-plane dipoles in neighboring unit cells. For example, in mode *D*_6_ we see that the distribution of the dipoles in the isolated quadrumer is antisymmetrical; when many quadrumers are considered, the fields generated by in-plane dipoles of the quadrumers in the bulk are perfectly balanced with opposite phase and destructively interfere with one another, so the bulk only weakly radiates to the far field. This phenomenon is less effective at the edges and the corners of the sample, allowing for a stronger radiation to the far field, and the corners and edges are observed bright. Lasing occurs over the whole sample as out-of-plane and near fields exist also in the bulk, as shown by our FEM simulations (see Figure 2); only the in-plane dipolar fields–that could provide far-field emission–are suppressed in the bulk by destructive interference, as shown by the in-plane dipole field calculations in Figure 3. The destructive interference is less efficient at the edges, therefore the boundaries are providing an additional leak channel for the quasi-BIC to radiate and be observed in the far field. This leakage mechanism is different from some previous BIC works^16,17^ where there are leakage mechanisms that couple out light only from the bulk of the sample.

The polarization of the light escaping from the edges is directly connected with the dipole orientation in each quadrumer, and by analyzing these polarization features we identify the lasing mode. For the case of horizontally filtered light in Figure 3a, the intensity maxima are located at the left and right edges of the array, whereas when a vertical filter is applied in Figure 3b, the intensity maxima are located at the top and bottom edges of the array. Our theoretical prediction confirms a strong emission from the same edges, resulting from a π-phase difference in the dipole orientation in the quadrumer. The case of antidiagonal filtered light is depicted in Figure 3c, where the top-left and bottom-right corners show the highest intensity, while the opposite corners are darker. Conversely, the diagonally filtered case in Figure 3d shows the opposite pattern, where the corners that are parallel to the orientation of the polarizer, that is, top-right and the bottom-left, appear brighter. The corresponding theoretical predictions present a strong emission from all edges of the sample, except at the opposite corners that are perpendicular to the orientation of the polarizer, in agreement with the observed experimental findings. Finally, in the case of left- and right-circularly polarized light in Figure 3e,f, we observe bright edges and corners, and no substantial difference between the two types of filters. A similar comparison of experiment and theory for the other seven modes does not produce agreement, thus, unambiguously identifying the lasing mode as *D*_6_.

**Figure 4:**
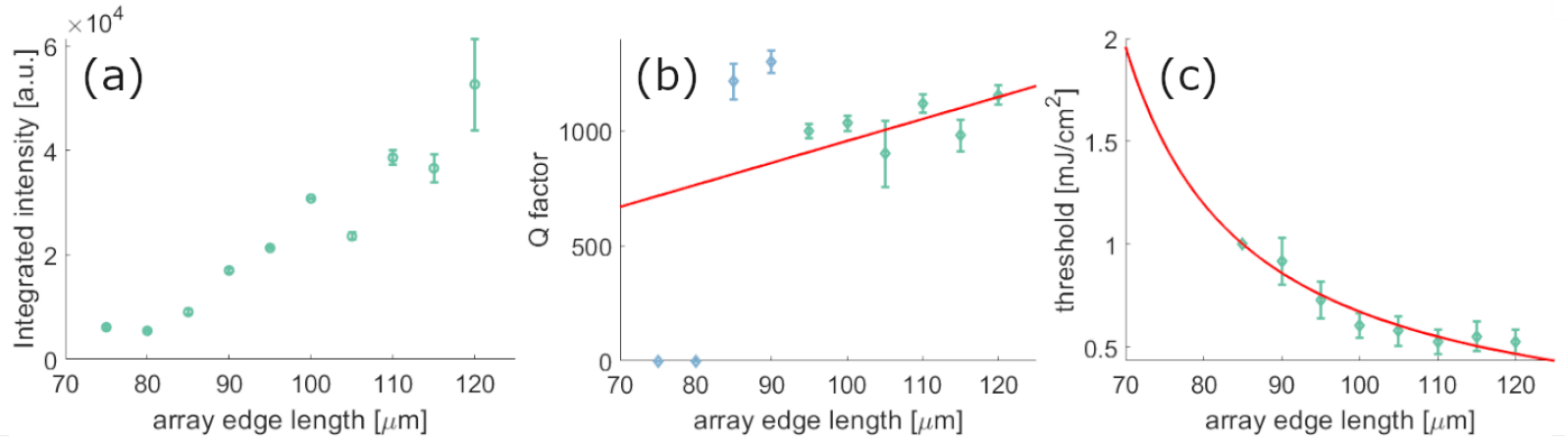
Integrated lasing peak intensities normalized by array size (a), *Q*-factors of the lasing emission peak for differently sized arrays at a pump fluence of 1.995 mJ/cm^2^ and lasing threshold for different array sizes (c). In (b) the green data points were included in a linear fit (red line), and in (g) the red line is a curve fit of the form 1/*x*. With decreasing array size, the peak intensity decreases and the threshold pump fluence increases. Arrays with an edge size below 80 μm did not show any lasing action.

With decreasing array size, the *Q*-factor of the quasi-BIC mode should decrease as the destructive interference in the bulk becomes less significant compared to the behavior near edges. In order to study the effect of the array size on the lasing action, we performed experiments on arrays ranging from 75 to 120 μm in size, see Supporting Information for details. We see that when less quadrumers are involved, the lasing features disappear gradually: the lasing peak integrated intensity decreases with decreasing array size until there is no lasing action observed in Figure 4a. In Figure 4b, we plot the *Q*-factors, as obtained from the lasing peak energy divided by its line width. These lasing peak *Q*-factors are decreasing with the edge length, except for two outlier points for which also the lasing peak intensity is small, and the spectrometer underestimates the peak-width. A much clearer trend is visible in the lasing threshold, which decreases as a function of the array size, see Figure 4c. This behavior can be explained and related to the *Q*-factor using a standard rate-equation model, as in ref (42). From this approach, one obtains that the lasing threshold value *N*_th_ is inversely proportional to the *Q*-factor of the lasing mode *N*_th_ = τω/(β_s_*Q*), where τ is the decay time between the two levels over which the inversion of population *N*_th_ is established, β_s_ is the coupling fraction of spontaneous emission to the lasing mode, and ω is the mode frequency; see the Supporting Information of ref (42) for more details. Given the linear dependence of the *Q*-factor on the array edge length, as seen in the red line of Figure 4 (b) at least for arrays with high intensity lasing peaks, then the threshold is expected to be inversely proportional to the array edge length, as also confirmed by the fit (red line) in Figure 4c.

We now comment on the differences between these corner features of the lasing mode in our system and the “corner modes” of higher-order topological insulators (HOTI). The HOTI are a new type of topological phase for which the boundary modes of a D-dimensional system exist in (D-2)-dimensions.^43^ A prototypical model is represented by the two-dimensional Su–Schrieffer–Heeger model (2D-SSH),^44−50^ which is constituted by a square lattice with four sites per unit cell, as our lattice in Figure 1. The first striking difference between the corner modes in HOTI and the corner feature of our BIC is that chiral symmetry in our platform is broken by the long-range radiative coupling between nanoparticles. Chiral symmetry is fundamental in HOTI and all models related to the BDI class, as it is the symmetry that protects the existence of the edge modes: by breaking it, the topological properties of the system can be modified and edge states eventually disappear.^51^ The second and most important difference is that, although our lattice is exactly that of the 2D-SSH model, we are in the topologically trivial regime (in the 2D-SSH sense). This is visible from Figure 1a, where the lattice terminates with a full unit cell, not with a “dangling” site. Remarkably, we see that our modes are independent of the edge termination, signaling a different mechanism than the conventional 2D-SSH tight-binding band model. The edge and corner features of the BIC mode seen in Figures 1–3 are explained from a destructive interference effect in the bulk arising from a field-circulation pattern of the lasing mode, not from a nontrivial Zak phase of the Bloch wave vectors of a Bloch Hamiltonian. Nonetheless, the BIC lasing mode has a topological origin, as we discuss in the following.

**Figure 5:**
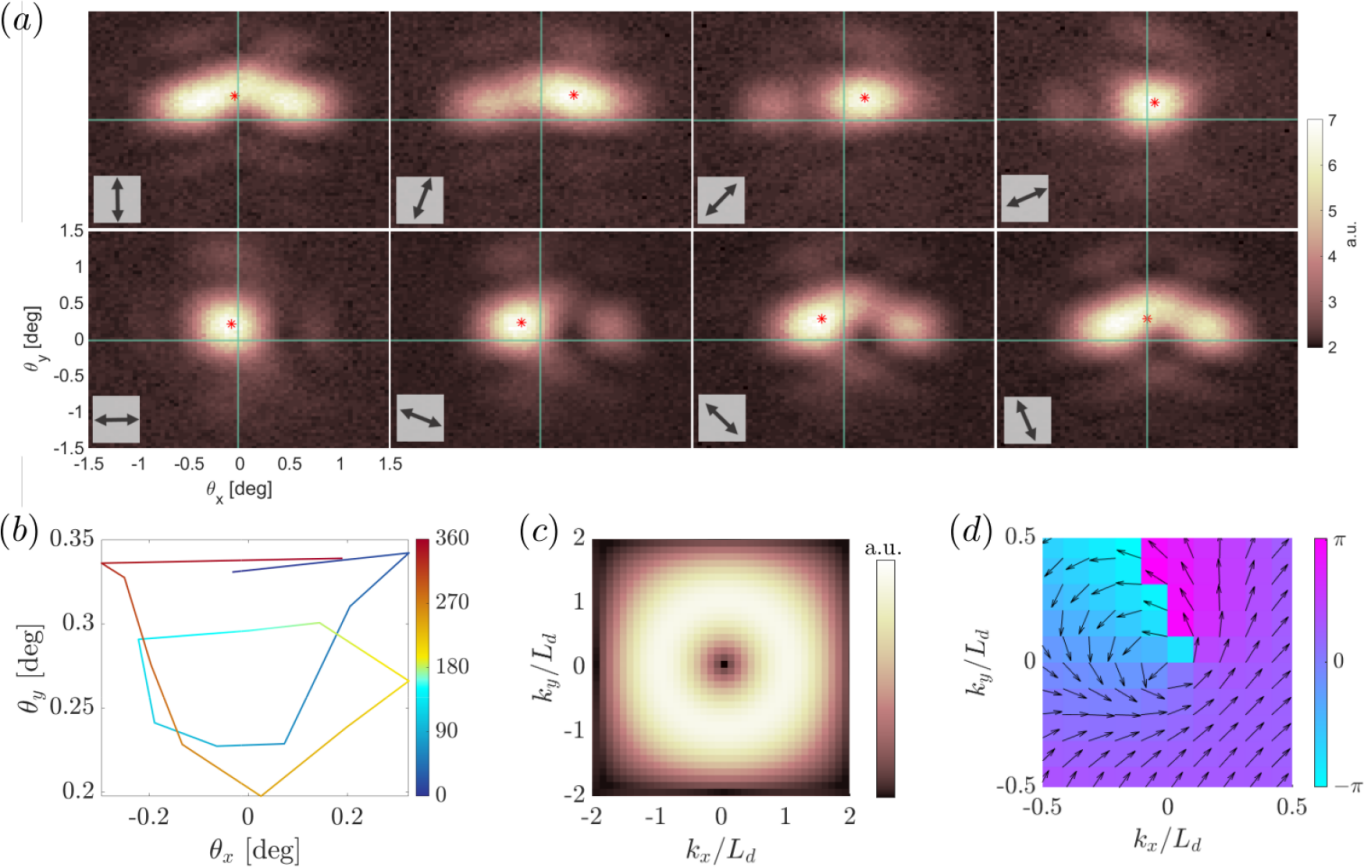
Polarization vorticity. (a) Momentum space images of the lasing emission (array with unit cell size of 590 nm, pumped with LCP pump at a fluence of 0.317 mJ/cm^2^), with different polarization filters in the detection path. The red dot marks the intensity center-of-mass. An overall clockwise rotation is evident when the polarization filter is rotated. (b) Intensity center-of-mass of the experimentally measured emission in *k*-space, the red dot in panel (a): a clockwise rotation is observed when the polarization filter is rotated from 0 to 360°. (c) Theoretical amplitude and (d) phase of mode *D*_6_’s far field in momentum space, calculated as the Fourier transform of the numerically calculated real space field (Figure 3) with no polarization filter.

In Figure 5a, we show the momentum-space intensity distribution of the lasing mode (pump fluence 0.317 mJ/cm^2^). In the detection stage, we apply different polarization filters that are oriented as indicated by the double-headed arrows in the insets. As we turn the polarization filter in a clockwise direction, we observe a gradual clockwise rotation of the momentum-space polarization, indicating that the lasing mode has a nontrivial vorticity in the far-field. To track this clockwise rotation, we computed the center-of-mass (COM) of the emission intensity, shown as a red dot in Figure 5a. A full rotation of the COM intensity trajectory in momentum space is observed in Figure 5b when the polarization filter is rotated by 180°, where the color indicates the given polarization angle. In the ideal case, the COM would be in the middle (the Γ-point) and remain inert, however, due to experimental imperfections, it is slightly off-centered and thus winds with the polarization rotation.

The topological nature of the BIC is numerically confirmed from the polarization vector winding in momentum space, following similar calculations as the ones introduced in ref (21); see Supporting Information. In Figure 5c,d, we show the amplitude and the phase of the far field of mode *D*_6_ in momentum space, calculated as the Fourier transform of the mode’s electric field with no polarization filter, from the finite-sized real-space images in Figure 3. The far-field vorticity is represented as a donut-shaped amplitude pattern centered on the Γ-point in Figure 5c. The absence of intensity in the center of the donut is due to the fundamental nature of a BIC, while the size of the donut reflects the particular mechanism that makes a BIC into quasi-BIC that can be observed in far field. In our case, fields coming from opposite sides of the donut at **k** and −**k** in momentum space correspond in real space to the edge and corner emission seen in Figure 3. In fact, the length of the system in real space *L* determines the size *L*_d_ = 2π/*L* of the donut in momentum space, see Supporting Information, Figure S7. The polarization vortex is evidenced in Figure 5d as a nonzero winding around the Γ-point of the polarization phase, whose topological charge is *q* = +1 (see Supporting Information). We note that the topological charge is a bulk quantity, therefore, it is defined in an infinite system. However, our quasi-BIC lasing mode is observable through the existence of the boundaries and is directly linked to the nontrivial topological charge of the bulk. The winding and topological charge of mode *D*_6_ were also computed from the FEM simulations in a system with periodic boundary conditions, yielding the same result for the polarization winding around the Γ point (see Supporting Information, Figure S2). These theoretical calculations in momentum space allow us to confirm the identification of the lasing mode with the quasi-BIC of topological charge *q* = +1, whose polarization pattern in real space is given by mode *D*_6_ in Figure 2.

## Conclusion

We have observed lasing in a quasi-BIC mode of a quadrumer nanoparticle array. The nature of the lasing mode was identified by combining theoretical mode analysis for a single quadrumer, numerical description of electromagnetic fields in both finite and infinite lattices, and polarization analysis of the experimental lasing emission. In infinite lattice simulations, the lasing mode in the lossless case shows typical BIC characteristics, while including losses reveals an interesting change in the momentum dependence of the *Q*-factor; namely, it has a minimum at normal incidence. The *Q*-factor nevertheless remains high, on the order of thousands, and the far-field emission is completely suppressed. In a finite system, emission occurs primarily at the edges and corners of the sample, both in theory and experiments.

Our quasi-BIC lasing mode has a topological charge *q* = +1, associated with polarization vorticity that winds once around the Γ-point in momentum space. The mode has a highly out-of-plane character. Previous work on out-of-plane BIC lasing in nanoparticle arrays used dielectric particles greater than 100 nm in height that support prominent out-of-plane modes.^17,34,35^ In our case, the metal particles are only 50 nm tall and the out-of-plane character of the mode results from the structure of the quadrumer. This demonstrates that particle complexes are amenable for designing a rich variety of out-of-plane modes, also using small nanoparticles that have reduced ohmic losses.

Our results reveal that multiparticle unit cells of nanoparticle arrays are a promising concept for realizing quasi-BIC modes whose darkness in the far-field is topologically protected against imperfections. Using this route, exceedingly high *Q*-factors are available, pushing strong light–matter interactions, lasing, and condensation phenomena to new regimes. The demonstrated feasibility of quadrumer array lasing inspires further explorations that are of general interest for topological photonics. What new phenomena may emerge when there is geometric frustration between the cluster and the main lattice? Can one observe lasing in modes with higher order topological charge? What would happen in a crossover from the radiative coupling case studied here to the tight-binding 2D SSH-model? This can be realized by bringing the particles closer to each other until near-field coupling dominates. Is the vorticity-related topological charge of the present case connected to the one originating from the chiral symmetry of the SSH model? Experimental studies of all these questions are feasible in the system introduced here, both in the lasing and nonlasing regimes.

## Supporting Information Available

*Q*-factor dependence on lattice plane momentum; Topological charge of the BIC as obtained from FEM simulations; Sample fabrication; Experimental setup; Calculation of the modes in the isolated quadrumer; Finite element method (FEM) simulations of the quadrumer lattice: dispersions, *Q*-factors, and far fields; Numerical calculations of the real space images of the lasing mode using fields generated by in-plane dipoles; Calculation of the polarization winding and topological charge; BIC lasing in the presence of defects; FEM simulation of vectorial in-plane field components and out-of-plane Poynting vector; Structural parameter dependence; Numerical calculations with different system sizes; Experiments with different array sizes (PDF)
