# Supplementary material for: Quasi-BIC Mode Lasing in a Quadrumer Plasmonic Lattice
Source: ACS Photonics. 2022 Jan 7;9(1):224–32. doi: 10.1021/acsphotonics.1c01416 (PMC8780794; doi:10.1021/acsphotonics.1c01416)
Supplement: Supplementary file 1 — ph1c01416_si_001.pdf [file ph1c01416_si_001.pdf]

Supporting Information

Quasi-BIC mode lasing in a quadrumer plasmonic lattice

Rebecca Heilmann[1], Grazia Salerno[1], Javier Cuerda[1], Tommi K. Hakala[2] and Päivi Törmä[1,*]

[1]Department of Applied Physics, Aalto University School of Science, P.O. Box 15100, Aalto, FI-00076, Finland
[2]Institute of Photonics, University of Eastern Finland, FI-80101 Joensuu, Finland
[*]paivi.torma@aalto.fi

Q-factor dependence on lattice plane momentum

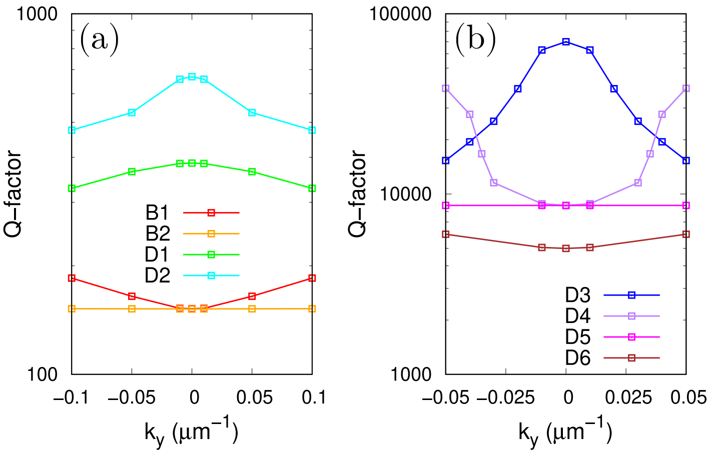

Figure S1: Q-factor dependence on lattice plane momentum (a) for modes polarized mainly in the lattice plane, and (b) for those polarized mainly out of plane, as calculated from FEM simulations.

Topological charge of the BIC as obtained from FEM simulations

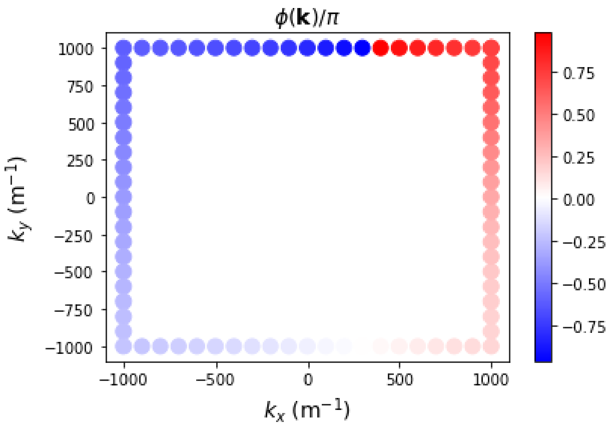

Figure S2: Winding of the polarization for the mode D_6, calculated by using the angle of the polarization vector: $\phi(\mathbf{k}) = \arg[\mathbf{p}(\mathbf{k}) \cdot \hat{x} + i\mathbf{p}(\mathbf{k}) \cdot \hat{y}]$, with $\mathbf{p}(\mathbf{k}) = (\hat{x} \cdot \langle \mathbf{u}_{\mathbf{k}}(\mathbf{r}, z)\rangle)\hat{x} + (\hat{y} \cdot \langle \mathbf{u}_{\mathbf{k}}(\mathbf{r}, z)\rangle)\hat{y}$, where $\mathbf{u}_{\mathbf{k}}$ is the electric field that is obtained from FEM simulations for a single unit cell with periodic boundary conditions, and $\langle \cdot \rangle$ means the spatial average over a surface $z = 5.4$ μm away from the lattice plane. The winding direction is unaltered by the choice of the plane for the spatial average. The topological charge is then calculated upon discretization of the formula: $q = \frac{1}{2\pi} \oint_C d\mathbf{k} \cdot \nabla_{\mathbf{k}} \phi(\mathbf{k}) \approx \frac{1}{2\pi} \sum_i (\phi(\mathbf{k}_{i+1}) - \phi(\mathbf{k}_i))$, along the chosen counterclockwise contour shown in the figure. The resulting winding and topological charge $q \simeq +1$ fully agree with the result in Fig. 5(d) and the observations in the main text.

Sample fabrication

The 100 x 100 μm^2 nanoparticle arrays are fabricated on a borosilicate substrate using electron beam lithography (EBL). A 200 nm thick Poly(methyl methacrylate) (PMMA) layer is spin-coated and baked on the glass substrate. After evaporating 10 nm aluminium on top, the PMMA is patterned using EBL. The aluminium is etched in a 1:1 mixture of de-ionized water and AZ351B developer, and the PMMA is developed in a 1:3 mixture of methyl isobutyl ketone and isopropanol. A 2 nm thick adhesive titanium layer and 50 nm of gold are evaporated. The excess PMMA and metal are removed in acetone lift-off.

The dye solution is index-matched to the substrate and consists of a 1:2 mixture of DMSO:Benzyl Alcohol. IR-140 dye molecules are dissolved in a 10 mM concentration. The dye solution is injected into a 0.8 mm thick press-to-seal silicone isolator chamber between the sample slide and an additional borosilicate slide. For transmission measurements, the arrays are immersed in index-matching oil and a cover slip is placed on top.

Experimental setup

A schematic of the experimental setup is shown in Figure S3. In transmission measurements, the sample is illuminated by white light from a halogen lamp, while for the lasing measurements the sample is excited with an external pump laser (800 nm central wavelength and a repetition rate of 1 kHz), which is left-circularly polarized and covered the whole sample area. The pump fluence is controlled with a neutral density wheel.

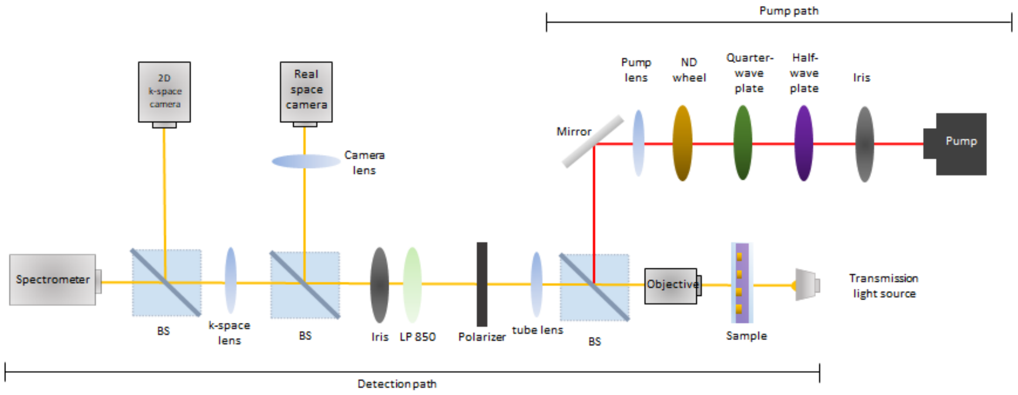

Figure S3: **Schematic of the experimental setup used in the lasing experiment.** The two CMOS cameras and the 2D CCD camera in the spectrometer allow for simultaneous measurements of angle-resolved spectra, real space images, and 2D k-space images; in this way one can confirm that the images correspond to the lasing mode (single narrow peak at the spectrometer). Here, ND, BS and LP 850 stand for neutral density, beam splitter and long pass filter with 850 nm cutoff length, respectively.

The transmitted or emitted light from the sample is collected by a 0.3 NA objective with a tube lens. The optional polarization filters are placed behind the tube lens to filter out single polarization states. In lasing measurements, an additional

850 nm long pass filter is used to filter out reflections of the pump beam. The back focal plane of the objective is focused onto the entrance slit of the spectrometer. Each point on the slit corresponds to an emission angle θ_y, which is related to the in-plane wavevector by $k_y = k_0 \sin(\theta_y)$ with $k_0 = 2\pi/\lambda_0$. Here, λ_0 is the free space wavelength. Thus, the 2D CCD camera inside the spectrometer resolves the angle of the light coming from the sample along one axis and the energy along the other. The real space images and 2D k-space images are collected by two separate CMOS cameras.

Calculation of the modes in the isolated quadrumer

We consider an isolated quadrumer for which the dipole moment on the i-th particle is $\mathbf{P}_i = (P_i^x, P_i^y)$. Particles are coupled to their neighbours in a polarization-dependent way, see Fig. 2(a), such that $\Omega_L = \langle \mathbf{P}_i \cdot \mathbf{L}_i | \hat{H} | \mathbf{P}_{i+1} \cdot \mathbf{L}_i \rangle$, when the dipoles are oriented longitudinally to the link connecting neighbouring particles i and $i+1$, while $\Omega_T = \langle \mathbf{P}_i \cdot \mathbf{T}_i | \hat{H} | \mathbf{P}_{i+1} \cdot \mathbf{T}_i \rangle$ for dipoles that are oriented transversely to the neighbouring link[1;2]; see notation in Fig. 2(b). In the basis $(P_1^x, P_1^y, P_2^x, P_2^y, P_3^x, P_3^y, P_4^x, P_4^y)$, the coupling matrix reads:

$$\hat{H} = \begin{pmatrix} \varepsilon & 0 & \Omega_L & 0 & 0 & 0 & \Omega_T & 0 \\ 0 & \varepsilon & 0 & \Omega_T & 0 & 0 & 0 & \Omega_L \\ \Omega_L & 0 & \varepsilon & 0 & \Omega_T & 0 & 0 & 0 \\ 0 & \Omega_T & 0 & \varepsilon & 0 & \Omega_L & 0 & 0 \\ 0 & 0 & \Omega_T & 0 & \varepsilon & 0 & \Omega_L & 0 \\ 0 & 0 & 0 & \Omega_L & 0 & \varepsilon & 0 & \Omega_T \\ \Omega_T & 0 & 0 & 0 & \Omega_L & 0 & \varepsilon & 0 \\ 0 & \Omega_L & 0 & 0 & 0 & \Omega_T & 0 & \varepsilon \end{pmatrix}, \tag{1}$$

where ε is a zero-point energy. In our case $\Omega_L \approx 0$ and Ω_T is finite; in this case we find two sets of four-fold degenerate modes, at energy $\approx \varepsilon \pm \Omega_T$. The spatial structure of the eigenmodes is shown in Fig. 2(c), where arrows correspond to the electric dipole moment orientation.

Finite element method (FEM) simulations of the quadrumer lattice: dispersions, $Q-$factors, and far fields.

In order to obtain the eigenmodes sustained by the quadrumer lattice, their corresponding dispersion relations (i.e., the energy dependence on the component of the $\mathbf{k}-$vector that is parallel to the lattice plane), and the $Q-$factors, we carried out finite-element simulations using the commercial software Comsol Multiphysics. We simulated an infinite square lattice by modelling a single unit cell of size $p_x \times p_y = 590 \times 590$ nm^2, and set periodic boundary conditions in the x- and y- directions. The metallic nanoparticles that form the quadrumer are realistically modelled as cylinders with a height of 50 nm and a diameter of 100 nm. The considered center-to-center distance of neighbouring nanoparticles is 147.5 nm. The optical response of the metallic nanoparticles is modelled using Johnson and Christy tabulated data for the complex permittivity of gold (Au).[3] A background refractive index of 1.52, accounting for the dye solution and substrate, is also considered.

The simulations provide a complex eigenfrequency associated with each mode: $\omega(k_y) = \omega'(k_y) + i\omega''(k_y)$, where k_y is the lattice plane (in-plane) wavevector. The dispersion relation is given by the real part $\omega'(k_y)$, whereas the presented $Q-$factor is obtained by calculating the ratio: $Q = \omega'(k_y)/\omega''(k_y)$. The far fields are obtained by averaging the in-plane electric field amplitude: $|\mathbf{E}_{||}| = (|\mathbf{E}_x|^2 + |\mathbf{E}_y|^2)^{1/2}$ over a monitor placed at a $XY-$plane, at a distance several lattice periods away from the lattice plane.

Numerical calculations of the real space images of the lasing mode using fields generated by in-plane dipoles

From the knowledge of the mode in-plane dipole orientation at each site of the quadrumer, we can calculate the diffraction pattern generated by the nanoparticle array, following the same procedure as done in Ref.[4]. Each nanoparticle, with electric dipole moment $\mathbf{P}_s$, is viewed as a monochromatic point source located at a position $\mathbf{R}_s$ of an Airy pattern. The electric field is $\mathbf{E}_s(\mathbf{r}) \propto \mathbf{P}_s \mathcal{J}_1(\alpha|\mathbf{r}-\mathbf{R}_s|)/(\alpha|\mathbf{r}-\mathbf{R}_s|)$, where α is an inverse-length parameter depending on the setup, and $\mathcal{J}_1$ is the first order regular Bessel function. The interference pattern is built-up as a sum of all the nanoparticles' contribution, such that the resulting intensity of the array is $I(\mathbf{r}) \propto |\sum_s \mathbf{E}_s(\mathbf{r})|$. In Figs. 3, the parameter α was chosen to be $\alpha = 1.5/p$, where p is the unit cell size. The exact choice of the value of α does not qualitatively affect the intensity pattern as long as the distance between image centres of two neighboring particles $|\mathbf{R}_{s_1} - \mathbf{R}_{s_2}| \ll 3.83/\alpha$ is well below the radius of the first Airy disk minimum. These calculations take into account the vectorial (in x-y plane) nature of the fields radiated by in-plane dipoles of the nanoparticles in the different modes. They do not involve out-of-plane dipoles or near fields, corresponding to light that does not radiate off from the sample plane; those are captured by the FEM simulations. Thus, complete destructive interference in the bulk of the sample in these in-plane dipole field calculations does not mean that there would be no light or lasing in the bulk in the experimental system: the actual lasing mode as obtained by FEM is largely of out-of-plane character and involves near fields, and can thus lase in the bulk. The in-plane dipole field calculations, however, describe correctly the emission to the far field by the different modes, as the far field emission is essentially determined by the orientations of the in-plane dipoles in the quadrumers.

Calculation of the polarization winding and topological charge

The electric field is obtained from FEM simulations as a function of the in-plane momentum $\mathbf{k}$ as $\mathbf{E}_\mathbf{k} = e^{i\mathbf{k}\cdot\mathbf{r}}\mathbf{u}_\mathbf{k}(\mathbf{r}, z)$. Following Ref.[5], the polarization vector is defined as $\mathbf{p}(\mathbf{k}) = (\hat{x} \cdot \langle \mathbf{u}_\mathbf{k}(\mathbf{r}, z)\rangle)\hat{x} + (\hat{y} \cdot \langle \mathbf{u}_\mathbf{k}(\mathbf{r}, z)\rangle)\hat{y}$, where $\langle \mathbf{u}_\mathbf{k} \rangle$ indicates the spatial average of the periodic part of the electric field $\mathbf{u}_\mathbf{k}$, over a surface defined by $\mathbf{r} = x\hat{x} + y\hat{y}$ for constant z away from the lattice plane. The winding of the polarization vector is calculated over a closed path $\mathcal{C}$ that encircles the BIC, i.e. $\mathbf{k} = 0$. The topological charge counts exactly how many times the polarization of $\mathbf{p}(\mathbf{k})$ winds around the BIC point and is defined to be an integer $q = \frac{1}{2\pi}\oint_\mathcal{C} d\mathbf{k} \cdot \nabla_\mathbf{k}\phi(\mathbf{k})$, where $\phi(\mathbf{k}) = \arg[\mathbf{p}(\mathbf{k}) \cdot \hat{x} + i\mathbf{p}(\mathbf{k}) \cdot \hat{y}]$. An alternative calculation of the polarization vector and BIC topological charge is obtained from the Fourier transform of the

real space in-plane dipole field calculations $(\mathbf{E}_x, \mathbf{E}_y)$ in Fig. 3. The polarization can be directly computed in momentum space as $\mathbf{p}(\mathbf{k}) \propto f(\mathbf{E}_x) + if(\mathbf{E}_y)$, where $f(\cdot)$ indicates the Fourier transform. The square modulus and the phase of $\mathbf{p}(\mathbf{k})$, obtained from this method, are shown in Fig. 5. The Fourier-transform calculation has the advantage of being computationally faster than FEM simulations, although losses are not realistically accounted for. However, the topological charge and the polarization winding calculated from these two approaches is the same, as can be seen in the Supplementary Figure S2.

BIC lasing in the presence of defects

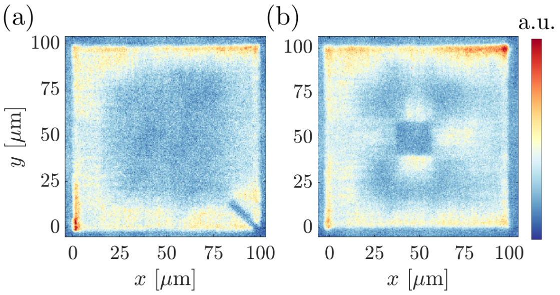

Figure S4: **Lasing mode robustness.** Real space intensities of the array with a (a) corner defect, and (b) central square defect. The defect is realised by removing nanoparticles in the area, which remains dark, in contrast to the edge and corners that still exhibit the lasing features.

As discussed in the main text, the BIC vorticity in the far-field polarization is defined in the bulk of the system as a non-trivial winding around the Γ point in momentum space, but is visible in real space due to the presence of edges. Specifically, the edges break the destructive interference made possible by the polarization pattern, such that the BIC emission is stronger at the edges and corners. We found that this phenomenon is independent of the selected edge termination, and is also robust to defects. We consider samples with a line corner and a central square defect realised by removing nanoparticles in the area, see respectively Fig. S4(a) and Fig. S4(b). In both cases we see that the edge and corner features, identifiable in the defect-free sample above the lasing threshold in Fig. 1(c) of the main text, are still clearly visible, indicating a topologically protected BIC.

FEM simulation of vectorial in-plane field components and out-of-plane Poynting vector

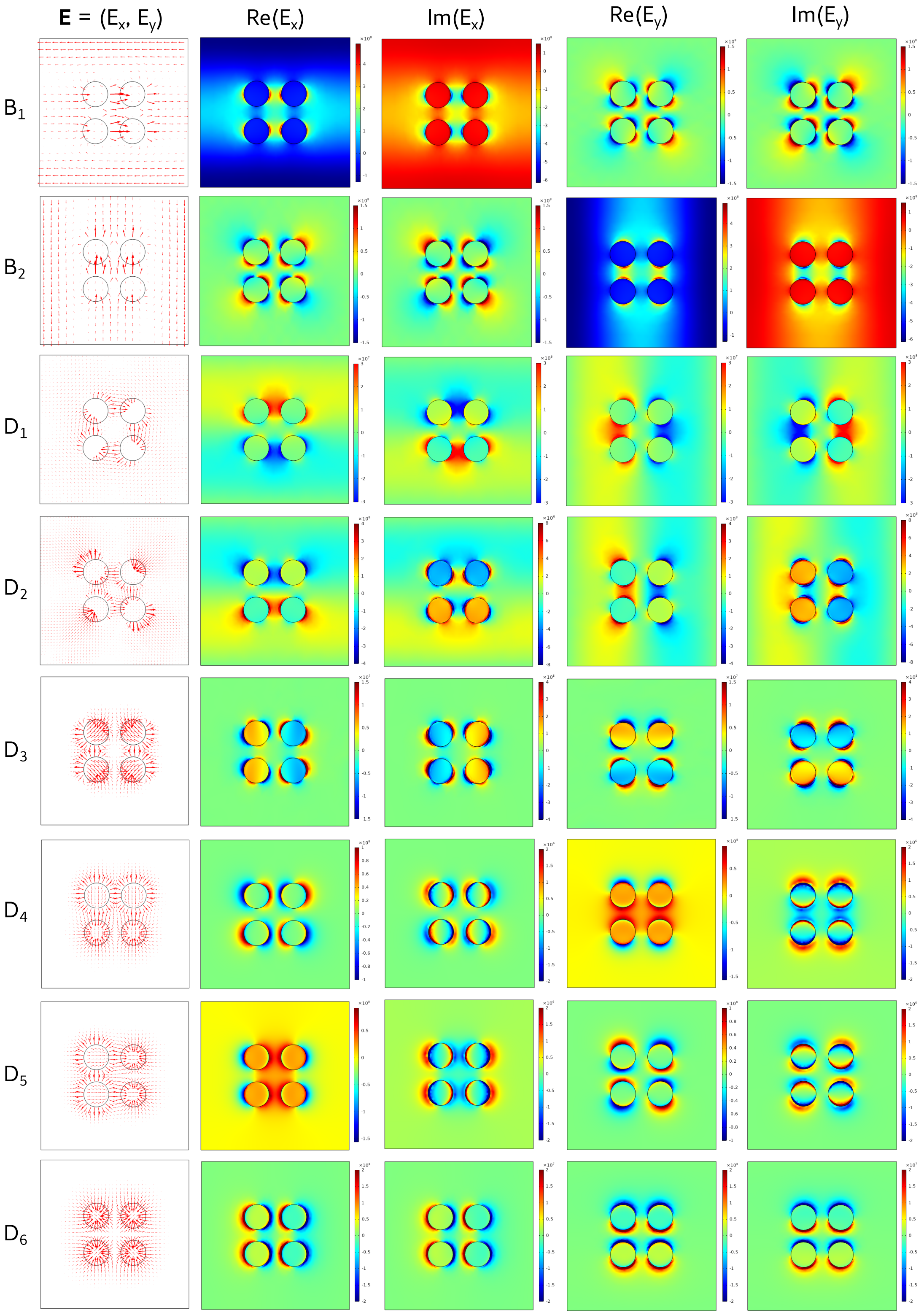

Figure S5: Vectorial in-plane electric field $\mathbf{E}$, and E_x, E_y field components (in V/m) of all the modes considered in Fig. 2(c) of the main text. The arrows are scaled in proportion to the field intensity. Only the relative values of the field amplitude (colorscale bars next to the E_x and E_y panels) are meaningful, and they are shown for comparing the relative magnitude of the different field components. These plots are depicted at the lattice plane, corresponding to the experimental surface of the glass substrate on which the nanoparticles sit.

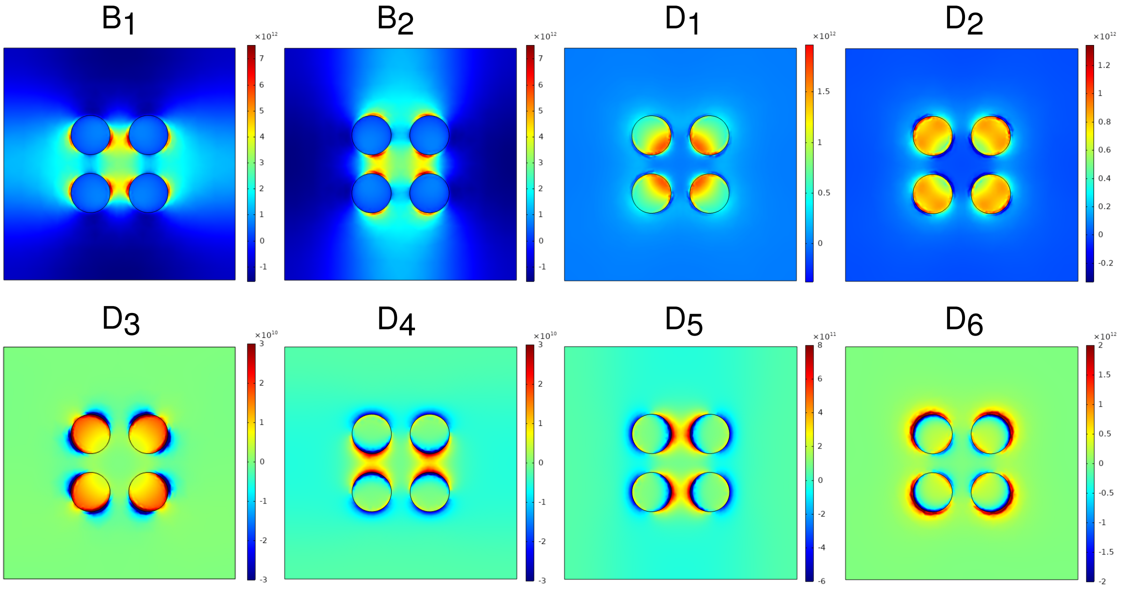

Figure S6: Out-of-plane component of the Poynting vector at the lattice plane (in W/m^2), for all the modes considered in Fig. 2(c) of the main text for an infinite lattice. The mode D_6 has both negative and positive contributions close to the nanoparticles which may cancel radiation to far field, as expected for BICs. We carried out analogous calculations at a XY plane located 8 μm away from the lattice plane, and we found that the out-of-plane component of the Poynting vector for the modes $B_{1,2}$ outgrows in four orders of magnitude that of the modes $D_3 - D_5$, and in seven orders of magnitude that of the modes D_1, D_2 and D_6. This is consistent with the results of Figs. 2(f),(g) of the main text.

Structural parameter dependence

In addition to the lattice presented in the main text, we studied lattices with different structural parameters as explained as follows. We studied array sizes of 50 μm x 50 μm, 75 μm x 75 μm, 100 μm x 100 μm, 150 μm x 150 μm and 200 μm x 200 μm. In addition, the distance of the particles in the quadrumer was changed, where β ranged from 0.46 to 1. As described in the text, the distance of the particles within the quadrumer is $a = \beta p/2$, i.e. in case of a lattice period of 590 nm, the center to center distance of the particles ranged from 135 nm to 295 nm. The arrays examined with varying sizes and β had a lattice constant of 590 nm. Further, the lattice constant was varied between 570 and 590 nm in 10 nm steps for 100 μm x 100 μm sized arrays and β=0.5.

The lattice constant did not affect the real space patterns of the lasing mode, i.e. the emission from the edges and corners from the array is clearly visible and show the same polarization dependence as the array shown in the manuscript. However, for a lattice constant of 570 nm the emission from the bulk of the array becomes comparable to the emission from the edges and the corners and we observe two lasing peaks. The lasing wavelength in these type of systems depends on the lattice constant as lasing is observed at the Γ-point. Therefore, changing the lattice constant changes the lasing wavelength. This, in turn, may lead to dramatic changes in the lasing behaviour (e.g., no lasing) since we always have the same molecule, and the lasing phenomenon depends crucially on where the lasing wavelength is located with respect to the emission-absorption spectrum of the molecule.

Arrays smaller than the one presented in the manuscript (100 μm x 100 μm) did not show any signature of lasing, whereas the larger ones did. The 150 μm x 150 μm large array showed more emission from the edges of the sample than from the bulk, whereas the 200 μm x 200 μm large array did not show any specific pattern in real space. The array size modifies the Q-factor of the modes, which is typically smaller for smaller arrays and therefore the array size affects the existence of lasing. A more detailed discussion on the effect of the array size is given in Sections "Numerical calculations with different system sizes" and "Experiments with different array sizes" below.

The interparticle distance of the particles in the quadrumer, i.e. β, has a large effect on the lasing ability of the structure. Arrays with $\beta > 0.58$ did not lase, whereas the samples with $0.46 < \beta < 0.56$ were lasing with the same lasing wavelength. The real space pattern in the unpolarized case was similar to the one presented in the manuscript. If the particles are further away from each other, the near field coupling within the particles in the quadrumer becomes weaker. At some distance, the collective quadrumer mode breaks down completely and the single particles are coupled via the lattice modes. For instance, if β=1, the array equals a square array with a lattice constant of 295 nm with one particle per unit cell, where lasing is expected at a different wavelength. Hence, arrays with $\beta > 0.58$, could be thought of as distorted square lattices, which might show lasing at different wavelengths as well. However, to verify this, a different molecule would need to be used.

Numerical calculations with different system sizes

The numerical calculations are to a great extent independent on the system size. In Fig. S7 we show how smaller arrays of 10-by-10 and 25-by-25 unit cells share the same real space features of the 50-by-50 array, especially regarding the edge features. Interestingly, as the system size is reduced, the intensity in the bulk slightly increases; this mechanism evidences how the destructive interference between the fields radiated by the neighbouring dipoles is less effective when the area of the bulk is reduced, thus we expect a decrease of the Q-factor as well; see next Section for experimental results. Nonetheless, the momentum space donut size directly depends on the real space length L of the system, as explained in the main text.

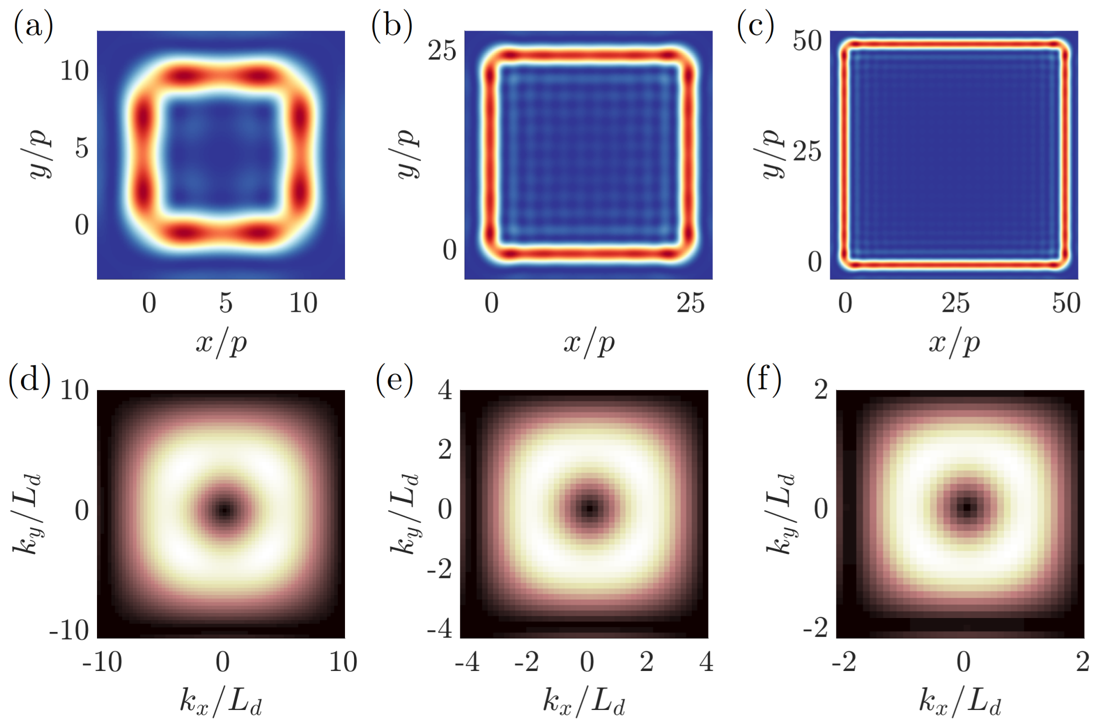

Figure S7: Calculation of the real space (a-b-c) and momentum space (d-e-f) intensities of the lasing mode, for different array sizes: 10-by-10 (a,d); 25-by-25 (b,e); and 50-by-50 unit cells (c,f). The momentum space images are rescaled with a common length that is the donut size of the 50-by-50 array $L_d = 2\pi/L_{50*50}$. As the size of the array in real space is halved or made 5 times smaller, the size of the donut in momentum space doubles or becomes 5 times bigger.

Experiments with different array sizes

We conducted experiments with different array sizes, where the array edge size was systematically changed in 5 μm steps from 75 to 120 μm. The pump spot size, the size of the iris and the exposure time of the spectrometer were kept constant during the measurements. For arrays that were lasing (i.e. edge length larger than 80 μm), the measurement was repeated five times and for arrays that were not lasing three times. To analyse the lasing peak intensity, the peak area of the spectrometer data

was integrated, i.e. the counts of each pixel were summed up. The integration area was kept constant for all measurements. Example threshold curves for each array size are shown in Fig. S8 (a) and normalized by the array size in Fig. S8 (b). It is clearly visible that the integrated intensity decreases, and the threshold pump fluence increases, for small array sizes. The integrated peak intensity normalized by the array size and the Q-factors in the lasing regime for a pump fluence of 1.995 mJ/cm^2 are shown in Fig. S8 (c) and (d) respectively. The Q-factor are calculated as the lasing peak energy divided by its linewidth. The laser linewidth is, according to the standard laser theory, directly proportional to the linewidth of the cavity (the bare mode without gain), minus a contribution from gain. For smaller array sizes one would expect the quasi-BIC mode Q-factor to decrease and therefore the lasing performance to degrade. One indicator of this would be an increase in the threshold fluence for smaller array sizes, another could be the decrease of the lasing peak Q-factor; both reflect a decrease of the Q-factor of the bare mode (the quasi-BIC).

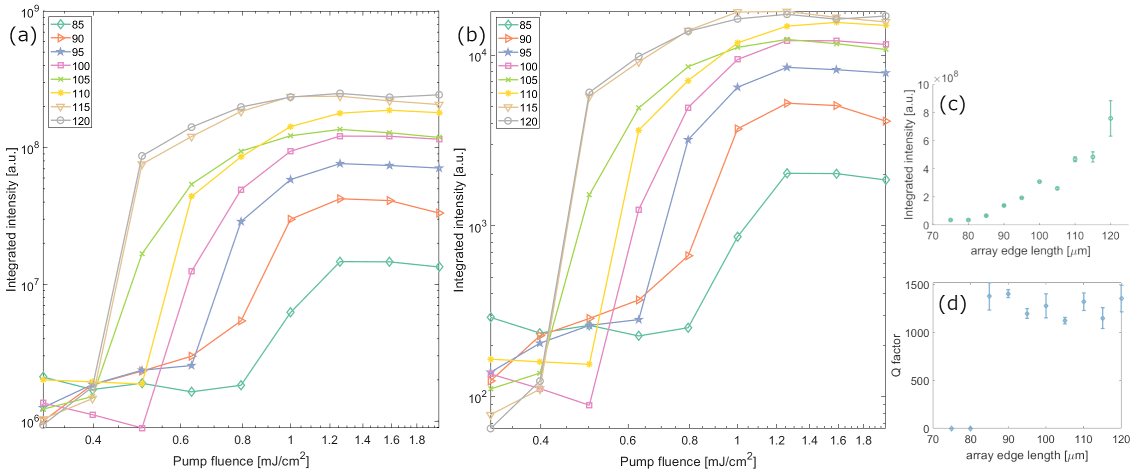

Figure S8: Threshold curves for different array sizes (a) and threshold curves for different array sizes normalized by array size (b), integrated lasing peak intensities at a pump fluence of 1.995 mJ/cm^2 (c), Q-factor at threshold for different array sizes (d). With decreasing array size, the peak intensity decreases and the threshold pump fluence increases. Arrays with an edge size below 80 μm do not show any lasing action. The Q-factor at threshold appears to be constant.

The lasing peak Q-factors for the arrays with edge lengths ranging from 95 to 120 μm are decreasing with the edge length. In Fig. 2 (b) of the main text it seems, however, that the Q-factors for the arrays with edge lengths of 85 and 90 μm have higher Q-factors. We attribute this to the behaviour of the spectrometer for low intensity peaks: If the intensity is very low, the counts of the pixels around the central peak are very low and comparable to the background. Hence, the peak is detected only on very few pixels and the peak appears to be narrow. For high intensity peaks however, the counts in the pixels around the central peak are much higher and distinguishable from the background and hence, the peak appears wider.

References

[1] VG Sala, DD Solnyshkov, I Carusotto, T Jacqmin, A Lemaître, H Terças, A Nalitov, Marco Abbarchi, E Galopin, I Sagnes, et al. Spin-orbit Coupling for Photons and Polaritons in Microstructures. Phys. Rev. X, 5(1):011034, 2015.

[2] Grazia Salerno, Alice Berardo, Tomoki Ozawa, Hannah M Price, Ludovic Taxis, Nicola M Pugno, and Iacopo Carusotto. Spin–orbit Coupling in a Hexagonal Ring of Pendula. New J. Phys., 19(5):055001, 2017.

[3] P. B. Johnson and R. W. Christy. Optical Constants of the Noble Metals. Phys. Rev. B, 6(12):4370, 1972.

[4] Rui Guo, Marek Nečada, Tommi K Hakala, Aaro I Väkeväinen, and Päivi Törmä. Lasing at K Points of a Honeycomb Plasmonic Lattice. Phys. Rev. Lett., 122(1):013901, 2019.

[5] B. Zhen, C. W. Hsu, L. Lu, A. D. Stone, and M. Soljačić. Topological Nature of Optical Bound States in the Continuum. Phys. Rev. Lett., 113:257401, 2014.
